# Assessing factors influencing communities’ acceptability of mass drug administration for the elimination of lymphatic filariasis in Guyana

**DOI:** 10.1371/journal.pntd.0009596

**Published:** 2021-09-20

**Authors:** Reza A. Niles, Charles R. Thickstun, Horace Cox, Daniel Dilliott, Clara R. Burgert-Brucker, Emma M. Harding-Esch, Nikita Clementson, Annastacia Sampson, Jean Seme Alexandre, Ana C. Morice Trejos, Ronaldo G. Carvalho Scholte, Alison Krentel

**Affiliations:** 1 Neglected Tropical Diseases Programme, Ministry of Health, Georgetown, Guyana; 2 School of Epidemiology and Public Health, University of Ottawa, Ontario, Canada; 3 Bruyère Research Institute, Ottawa, Ontario, Canada; 4 Global Health Division, RTI International, Washington DC, United States of America; 5 Clinical Research Department, London School of Hygiene & Tropical Medicine, London, United Kingdom; 6 Neglected, Tropical, and Vector Borne Diseases, Pan American Health Organization, Washington DC, United States of America; University Hospital Bonn, GERMANY

## Abstract

**Background:**

Guyana is one of four countries in the Latin American Region where lymphatic filariasis (LF) remains endemic. In preparation for the introduction of a new triple drug therapy regimen (ivermectin, diethylcarbamazine, and albendazole (IDA)) in 2019, an acceptability study was embedded within sentinel site mapping in four regions to assess mass drug administration (MDA) coverage and compliance, acceptability, and perceptions about treatment and disease. The results from this survey would inform the rollout of IDA in Guyana in 2019.

**Methods:**

Data collection for the study occurred in August 2019, using a validated questionnaire administered by trained enumerators. Across all regions, a total of 1,248 participants were sampled by the Filarial Mapping team. Four-hundred and fifty-one participants aged over 18 years were randomly selected for participation in an expanded acceptability questionnaire. All data were captured in Secure Data Kit (SDK).

**Results:**

Acceptability was measured using a mean acceptability score. Unadjusted mean scores ranged from 24.6 to 29.3, with 22.5 as the threshold of acceptability. Regional variation occurred across many indicators of interest: self-rated understanding about LF, mechanisms of LF transmission, LF drug safety and history of treatment during MDA. Region IV (Georgetown) recorded higher knowledge about LF, but lower compliance and acceptability. Number of pills was not perceived as a concern.

**Conclusion:**

Acceptability of MDA was good across all four regions under study. Results from this study set a baseline level for key indicators and acceptability, from which the acceptability of IDA can be measured. Regional variations across indicators suggest that localized approaches should be considered for social mobilization and MDA delivery to capture these contextual differences.

## Author summary

Guyana is one of four countries in the Latin American Region where lymphatic filariasis (LF) remains endemic in the community. In preparation for a new treatment regimen using three drugs instead of two, the research team assessed how community members in four regions of Guyana accepted the current two-drug treatment offered for LF during mass drug administration. Results from 390 participants showed that people generally accepted the treatment; however regional variation was high. Recommendations were made to the government program to inform the use of the new three-drug treatment regimen, including the need for localized approaches.

## Introduction

Lymphatic filariasis (LF), a neglected tropical disease (NTD), is a parasitic infection caused by the filarial nematodes *Wuchereria bancrofti*, *Brugia malayi*, and *Brugia timori* [1]. These parasites are transmitted by mosquito and mature into adult worms within the human lymphatic system following infection, causing excessive and painful swelling of the limbs, the genitals and, occasionally in women, the breasts [1–3]. Symptomatic LF can be debilitating, leading patients to suffer negative physical, mental, social and economic outcomes [4–6]. In 2019, LF was endemic in 72 countries and considered a public health threat in 50, representing a total at-risk population of approximately 858.3 million people still requiring preventive chemotherapy [7]. This represents a decrease of 43% from the total population identified as being at-risk for infection by the Global Programme for the Elimination of Lymphatic Filariasis (GPELF) since 2010 [7].

Efforts undertaken by the GPELF to eliminate LF as a public health problem are based on the strategies of mass drug administration (MDA) of preventive chemotherapy to break transmission of LF, and morbidity management and disability prevention (MMDP) to address LF-associated morbidities in infected patients [8–10]. Since 2000, the GPELF, national ministries of health, the World Health Organization (WHO) and other partners have worked together to implement these strategies with the goal of achieving the target of global elimination of LF as a public health problem [8,11,12]. Elimination is based on preventing infection through MDA, whereby all eligible community members living within an endemic area, regardless of infection status, are given an annual regimen of antifilarial treatments (diethylcarbamazine (DEC) plus albendazole (DA); or ivermectin plus albendazole (IA) in sub-Saharan African communities co-endemic with onchocerciasis) [10]. Annual treatment with DA or IA for at least five years has been shown to drastically reduce human reservoirs of *W*. *bancrofti*, *B*. *malayi*, and *B*. *timori*, preventing these parasites from being transmitted by mosquito vectors [10].

Guyana is one of four countries in the Latin America and Caribbean (LAC) Region known to be endemic with LF; with the others being Brazil, the Dominican Republic, and Haiti [13]. Risk-mapping of the environmental suitability for LF transmission in Central and South America has indicated that the physical environment in this region is conducive for widespread active transmission of *W*. *bancrofti* [14]. However, in LAC countries such as Guyana, transmission of the disease-causing parasite occurs in isolated foci [14]. One possible explanation is that transmission in the LAC region is highly influenced by various historical socioeconomic and sociodemographic risk factors, rather than environmental factors alone [14]. Such risk factors include poverty, living environment (urban slums and poor rural communities), age, gender, and ethnicity [5,14,15]. In addition, improvements in vector control, and water, sanitation, and hygiene (WASH) practices have limited widespread transmission of LF [14].

While active transmission is thought to be largely constrained to urban and poor socioeconomic environments, in 1995 approximately 81.3% of Guyana’s 799,000 people were estimated to be at-risk of LF infection [16]. Metrics provided by WHO in 2018 revised the total at-risk population in Guyana requiring MDA to 719,312 [17]. Nationwide mapping of LF infection among school children conducted in 2001 estimated approximately 9.3% of the population was positive for antigenemia, with variation across administrative regions [18]. The mapping revealed higher LF prevalence in six of Guyana’s ten administrative regions, especially in urban centers such as the cities of Georgetown, New Amsterdam and Linden [18]. The six regions with the highest prevalence of LF are also Guyana’s most populated [18].

To address LF endemicity in Guyana, the Guyanese Ministry of Health established the Lymphatic Filariasis Elimination Program. The program involved integrating treatments for lymphedema into primary healthcare services and a two-phase prevention effort [18]. Phase one of this prevention effort ran from 2003–2007 and involved social mobilization, and the promotion and distribution of DEC-fortified salt rather than antifilarial tablets, as the limited availability of health staff across various regions interfered with the implementation of a tablet-based MDA [18,19]. Phase one achieved much early success. However, due to disruptions in production and other technical problems, prevention efforts shifted to MDA with DA, signifying the beginning of phase two in 2008 [18,19]. While the program has achieved effective coverage in several regions, it has had variable success in others. As of 2017, approximately 60.9% of the population requiring preventive chemotherapy received it from the LF program [20]. In 2019, the LF program in Guyana adopted the use of the triple drug therapy for LF [ivermectin, DEC, and albendazole (IDA)] to accelerate its LF elimination program [21]. With the introduction of IDA, the LF program in Guyana also saw an opportunity to evaluate past coverage and compliance and revise social mobilization strategies prior to the launch of the new treatment regimen.

Evaluation of past coverage and compliance with MDA in Guyana was based on known factors influencing participation from global research [22]. In addition to coverage and compliance, increasingly individuals’ perceptions of the acceptability of interventions for LF elimination (e.g. MDA) are being investigated as an important consideration for their success [23]. Treatment acceptability is the extent to which people consider an intervention to be appropriate, based on their cognitive or emotional responses to that intervention [24]. Elements of a treatment that are thought to contribute to individuals’ evaluations of its acceptability include the treatment’s effectiveness, importance, intrusiveness, characteristics, side effects, and whether it aligns with the evaluator’s values or beliefs [24–31]. In addition, an individual’s sociodemographic or socioeconomic characteristics and the societal norms of the community to which they belong can influence their perceptions of the treatment’s acceptability [24–32]. If individuals or communities deem MDA to be unacceptable, they are unlikely to be motivated to participate in the intervention.

The purpose of this study was to assess Guyanese community members’ perceptions of the acceptability of MDA in four administrative regions through use of a community-based treatment acceptability assessment survey [23], embedded within a larger sentinel site survey. While coverage and compliance data are critical indicators of the program’s success, they may not adequately capture individuals’ attitudes towards the intervention or their motivations for participating (or not participating) in MDA. Furthermore, in regions or communities where coverage and compliance rates are high, the binary nature of these outcome variables often do not provide the granularity necessary for identifying reasons of non-compliance. The use of an acceptability measure allows for a more nuanced understanding of individuals’ attitudes towards MDA and can provide useful information about what aspects of the intervention encourage or deter individuals from accepting treatment. The acceptability study was used to inform the rollout of the 2019 MDA with IDA and provide recommendations to the LF program in Guyana to maximize success.

## Methods

### Ethics statement

Ethics was approved through the Ministry of Health, Guyana (IRB #599/2019) and through the Pan American Health Organization (PAHOERC #0090.01). Formal written consent was obtained for all participants.

### Study design & setting

**Fig 1:**
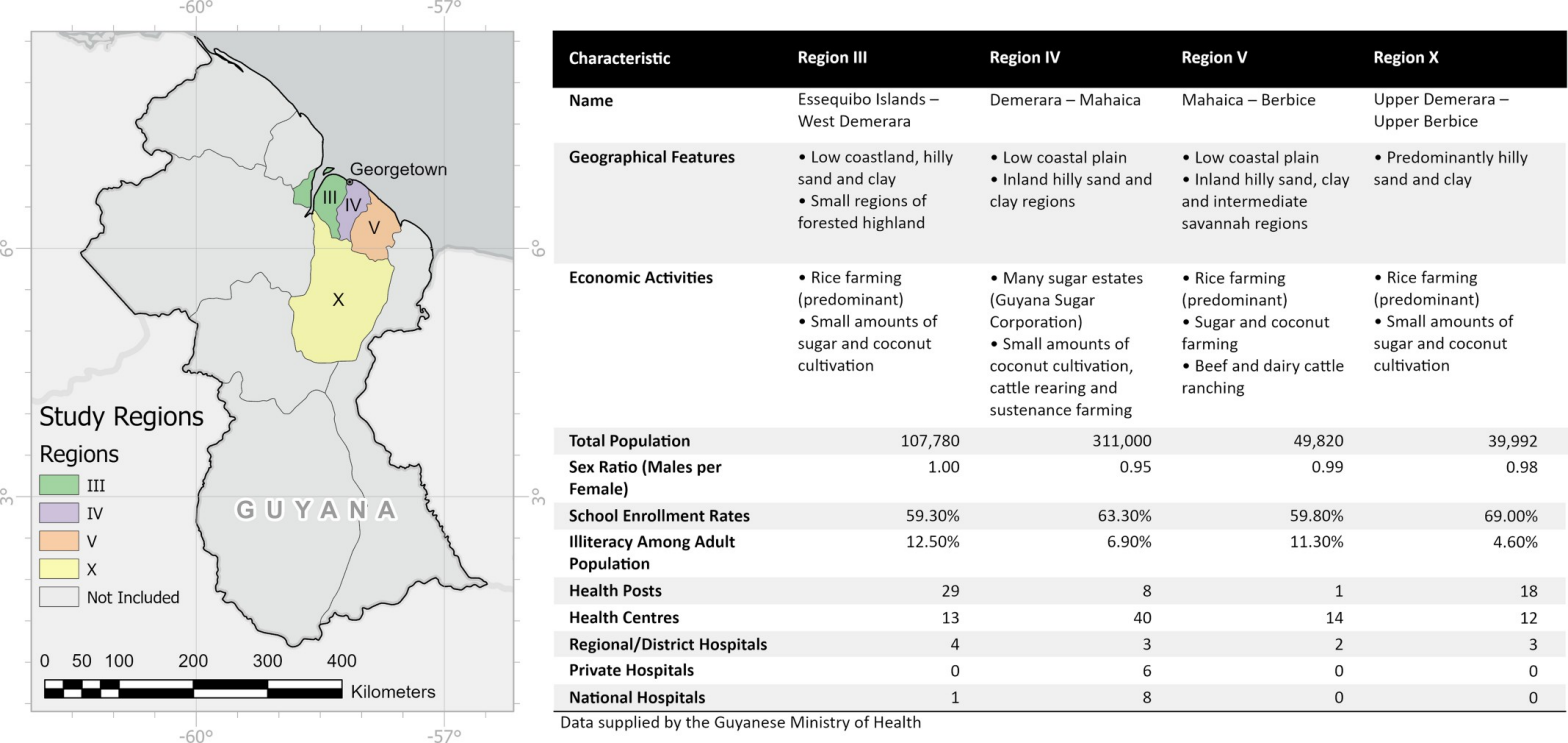
Description of study regions. Map content was produced with Esri ArcGIS software using data provided by Natural Earth (2018). [digital geospatial data]. Available online: https://www.naturalearthdata.com/ [17/08/2021].

A cross-sectional survey of four regions in Guyana was conducted as part of a Filarial Re-Mapping Project by the Guyanese Ministry of Health as recommended by GPELF and WHO to monitor filarial transmission in high-risk areas and to assess the baseline prevalence of LF in four targeted regions before the 2019 MDA using IDA (see Fig 1). Sampling of the mapping census was conducted according to WHO guidance on the monitoring and epidemiological assessment of MDA [33]. Sentinel sites were chosen from areas within the four regions using the following criteria: 1) populations of at least 500 people, targeting samples of at least 300 individuals; 2) where a high level of transmission was known to occur, or where difficulty in achieving high drug coverage was anticipated; and 3) that have a stable population unlikely to be affected by migration and that is similar in demographic characteristics to the implementation unit as a whole. Households were selected within the sentinel sites using Expanded Program on Immunization recommended methods [34].

To create a similar baseline characteristic of population acceptability related to MDA, an acceptability component was added to a random subsample of those included in the survey. A sample of 400 individuals across all regions was targeted in accordance with established methods for exploratory studies [35].

### Study population, participants & sampling

**Fig 2:**
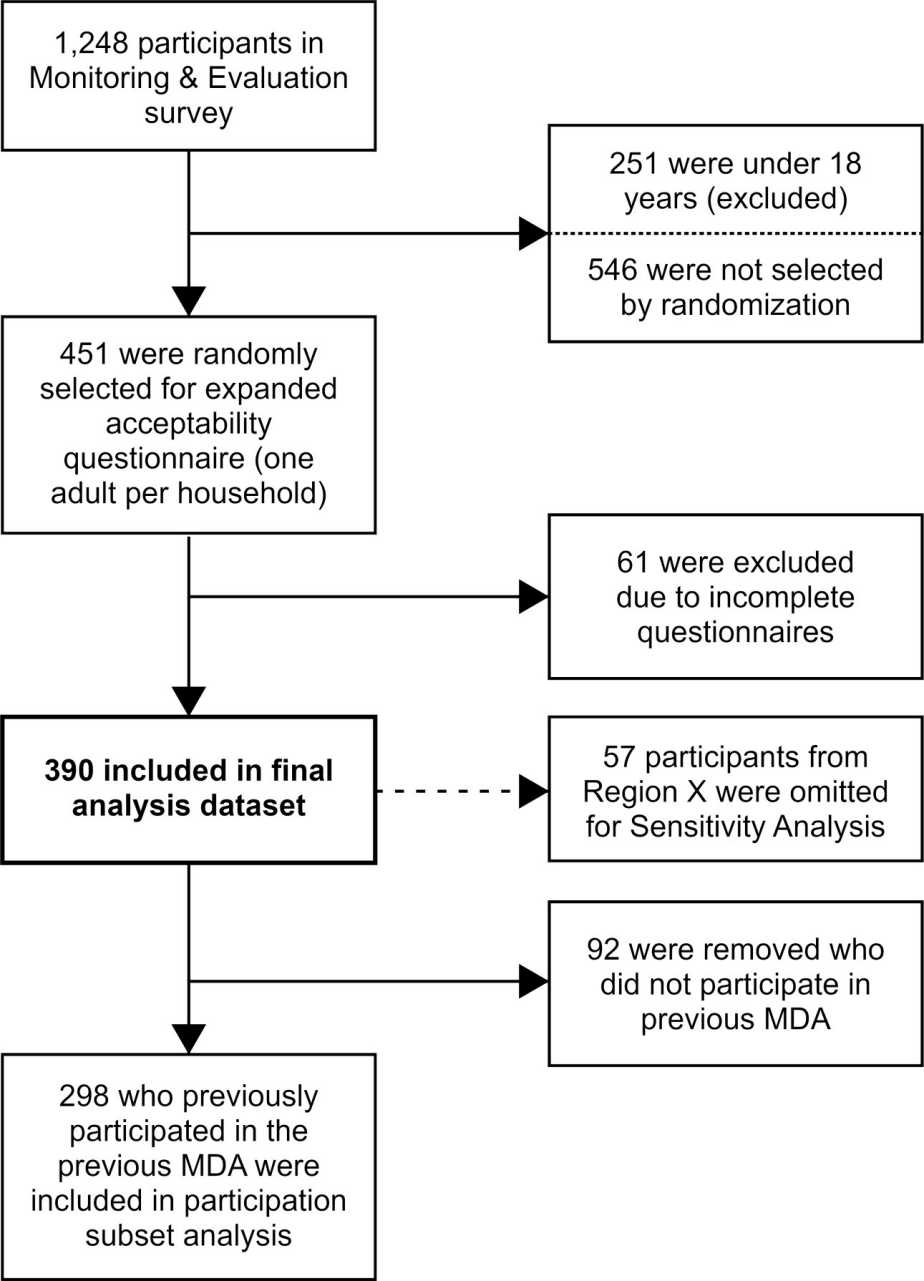
Acceptability study sampling frame and subset criteria.

Data collection for the study occurred between the 16^th^ and 31^st^ of August 2019, with one sampling team responsible for each study region. An outline of the sampling frame and further inclusions and exclusion for subgroup analyses is detailed in Fig 2. Across all regions, a total of 1,248 participants were sampled by the Filarial Mapping team. Of these, one participant over 18 years in each household was selected to receive an expanded acceptability questionnaire (n = 451) based on a previously validated tool [23]. Selection was determined by Random Number Generator [36] from among household members over 18 years of age who were present at the time of the survey. All data were captured on smart phones using the Secure Data Kit platform (Atlanta, GA, USA).

Three subsets of this dataset were used for analyses included in this paper. Our primary analysis dataset of 390 participants included those selected for the expanded acceptability questionnaire who responded to all our covariates of interest, and to at least five out of nine acceptability scoring questions [23]. Participants with four or fewer missing acceptability scoring questions had minimum values imputed for those questions as a conservative measure. No other variables were imputed.

Due to an unexplained high number of missing responses to key analysis variables in one of our study regions (Region X), a sensitivity dataset of 333 participants was created which excluded the remaining data in that region. Identical models were created for both the standard and the sensitivity dataset to assess whether the missing data were biased in a meaningful way that could impact the analyses.

A third subset of 298 participants was created to explore the attitudes of those who participated in the 2018 MDA of DEC and albendazole. This dataset included only those that self-reported as having swallowed all the pills provided to them during the 2018 MDA.

### Statistical analyses

Acceptability of DA was measured through a composite acceptability score which summed the values of nine acceptability indicators that were each scored across a four-point scale [23]. The possible range of acceptability scores were from 9–36, with 22.5 considered as the threshold of acceptability. The acceptability measure was derived from the Intervention Rating Profile tool [25] and has been described in more detail in a similar study in five countries [23]. The acceptability score was used as the primary outcome for analysis. A secondary analysis of variables previously shown to be implicated in treatment acceptability, such as knowledge levels, perceptions of the disease, and perceptions of the treatment, was conducted using Stata 14. Chi-square tests or Fisher’s exact tests were used where appropriate to compare these key variables across regions, sex, and previous compliance with treatment.

Given the importance of region as a covariate in the main study model, *post-hoc* individual analyses were conducted by region to determine if specific differences in factors associated with DA acceptability could be identified by region, to further target future MDA efforts. Due to the small sample size in the region-specific models (n = 111, 106, 116, and 57 for Regions III, IV, V, and X respectively), these should only be used to highlight areas for future research.

### Model building

Modeling analyses were conducted in SAS v9.4 using PROC GENMOD (SAS, 2012). Relevant covariates were selected based on prior analyses conducted by the research team in other settings. The Primary, Sensitivity, Compliance, and Regional models were all computed as standard linear regressions using the identity link function with the composite acceptability score as the response variable. The systematic non-compliance model was defined as a logistic regression with self-reported never/ever been treated in an MDA as the response variable. The term “systematic non-compliance” is used to describe individuals who self-reported that they had never been treated for LF during past rounds of MDA. Each of these models followed the same model building process: 1) univariable models were computed for each covariate of interest; 2) a full model was created using all covariates with significant responses (p < 0.05) in the univariable model, in addition to any variables that were believed to be possible confounders; and 3) covariates were removed from the model iteratively by increasing the level of significance until only significant covariates remained, unless doing so would cause a reduction in model fit as determined by Akaike information criterion (corrected) (AICc). AICc is a measure of model fit which controls maximum likelihood for the number of parameters included to prevent model overfitting, even in small samples [37].

## Results

### Sample population characteristics

A total of 390 surveys were included in the principal analyses. The sample data and regional breakdown are shown in Table 1. The response sample was heavily skewed towards females (72.8%). Similar sex ratios were observed across all four regions. The mean age of the sample population was 43.4 years (range 18–89 years). Most of the study sample (64.6%) had attained a secondary school education or greater, while only seven respondents indicated they had received no formal education. One third of survey participants elected to report and elaborate on their primary source of income using the “other” option. However, 22.3% of study participants classified their primary source of income as “private employment.” Agricultural activities, which includes fishing and farming, made up 17.2% of responses, largely driven by Region V which predominately reported this category. Mean acceptability scores ranged from 24.6 in Region III to 29.3 in Region V. All four regions were above the threshold of acceptability (a score of 22.5).

**Table 1:** Primary study population and key variables in total and subset by region.

Variable	Region III (n = 111)		Region IV (n = 106)		Region V (n = 116)		Region X (n = 57)		Total (n = 390)		pvalue
	Frequency	%	Frequency	%	Frequency	%	Frequency	%	Frequency	%	
Sex											
Male	34	30.6%	31	29.2%	25	21.6%	16	28.1%	106	27.2%	0.4275
Female	77	69.4%	75	70.8%	91	78.4%	41	71.9%	284	72.8%	
Age											
18–25	22	19.8%	16	15.1%	15	12.9%	10	17.5%	63	16.2%	0.5535
26–35	18	16.2%	15	14.2%	29	25.0%	9	15.8%	71	18.2%	
36–45	28	25.2%	23	21.7%	21	18.1%	12	21.1%	84	21.5%	
46–55	24	21.6%	22	20.8%	26	22.4%	12	21.1%	84	21.5%	
56 +	19	17.1%	30	28.3%	25	21.6%	14	24.6%	88	22.6%	
Education level attained											
No school at all	0	0.0%	1	0.9%	5	4.3%	1	1.8%	7	1.8%	<0.0001
Completed primary school	25	22.5%	9	8.5%	46	39.7%	12	21.1%	92	23.6%	
Completed middle school	13	11.7%	17	16.0%	5	4.3%	2	3.5%	37	9.5%	
Completed secondary school	65	58.6%	64	60.4%	55	47.4%	23	40.4%	207	53.1%	
Completed college/university	8	7.2%	14	13.2%	4	3.4%	19	33.3%	45	11.5%	
Other	0	0.0%	1	0.9%	1	0.9%	0	0.0%	2	0.5%	
Primary source of income											
Fishing or farming (agriculture)	3	2.7%	2	1.9%	62	53.4%	0	0.0%	67	17.2%	<0.0001
Daily laborer	19	17.1%	7	6.6%	2	1.7%	4	7.0%	32	8.2%	
Small scale enterprise	5	4.5%	10	9.4%	3	2.6%	5	8.8%	23	5.9%	
Private employment	16	14.4%	47	44.3%	3	2.6%	21	36.8%	87	22.3%	
Government/civil servant	5	4.5%	17	16.0%	7	6.0%	22	38.6%	51	13.1%	
Other	63	56.8%	23	21.7%	39	33.6%	5	8.8%	130	33.3%	
Self-rated understanding of LF											
No knowledge (1)	29	26.1%	18	17.0%	30	25.9%	2	3.5%	79	20.3%	<0.0001
A little (2)	24	21.6%	19	17.9%	40	34.5%	26	45.6%	109	27.9%	
Average (3)	35	31.5%	27	25.5%	17	14.7%	13	22.8%	92	23.6%	
Good (4)	6	5.4%	18	17.0%	18	15.5%	11	19.3%	53	13.6%	
Very good (5)	17	15.3%	24	22.6%	11	9.5%	5	8.8%	57	14.6%	
Mechanism of transmission											
Worms	7	6.3%	8	7.5%	19	16.4%	4	7.0%	38	9.7%	0.0391
Mosquitoes	88	79.3%	96	90.6%	94	81.0%	53	93.0%	331	84.9%	0.0220
Water	8	7.2%	11	10.4%	2	1.7%	5	8.8%	26	6.7%	*0.0326
Hereditary	0	0.0%	0	0.0%	1	0.9%	0	0.0%	1	0.3%	*1.0000
Other	4	3.6%	2	1.9%	0	0.0%	2	3.5%	8	2.1%	*0.1358
Don’t Know	21	18.9%	4	3.8%	23	19.8%	3	5.3%	51	13.1%	0.0003
Believes LF to be asymptomatic											
Yes	57	51.4%	67	63.2%	52	44.8%	32	56.1%	208	53.3%	0.0002
Maybe	27	24.3%	7	6.6%	17	14.7%	2	3.5%	53	13.6%	
No	6	5.4%	15	14.2%	18	15.5%	12	21.1%	51	13.1%	
Don’t know	21	18.9%	17	16.0%	29	25.0%	11	19.3%	78	20.0%	
Perception of number of people in village with LF											
None (1)	31	27.9%	9	8.5%	63	54.3%	16	28.1%	119	30.5%	†
Few (2)	10	9.0%	26	24.5%	7	6.0%	3	5.3%	46	11.8%	
Some (3)	3	2.7%	9	8.5%	7	6.0%	1	1.8%	20	5.1%	
Quite a lot (4)	0	0.0%	8	7.5%	2	1.7%	2	3.5%	12	3.1%	
Many (5)	0	0.0%	12	11.3%	1	0.9%	0	0.0%	13	3.3%	
Don’t know	67	60.4%	42	39.6%	36	31.0%	35	61.4%	180	46.2%	
Personal concern about LF											
No, not at all (1)	5	4.5%	1	0.9%	3	2.6%	1	1.8%	10	2.6%	†
Not really (2)	8	7.2%	6	5.7%	5	4.3%	1	1.8%	20	5.1%	
Maybe (3)	2	1.8%	10	9.4%	20	17.2%	2	3.5%	34	8.7%	
Yes, a bit (4)	27	24.3%	26	24.5%	39	33.6%	32	56.1%	124	31.8%	
Yes, definitely (5)	57	51.4%	63	59.4%	43	37.1%	18	31.6%	181	46.4%	
Don’t Know	12	10.8%	0	0.0%	6	5.2%	3	5.3%	21	5.4%	
	**Mean**	**SD**	**Mean**	**SD**	**Mean**	**SD**	**Mean**	**SD**	**Mean**	**SD**	
Acceptability Score	24.63	± 3.78	26.68	± 3.20	29.28	± 3.97	27.05	± 2.91	26.92	± 3.98	

* Denotes Fisher’s Exact Test.

† Data is too sparse across categories on these questions to run significance tests.

### Predictors of acceptability in the primary study population

Multivariable analysis (Table 2) indicated that region was an important predictor of acceptability, with Regions IV, V, and X having higher acceptability scores than those in Region III (1.22; p = 0.0038, 3.95; p < 0.0001, and 1.40; p = 0.0052 points higher, respectively). Individuals who had knowledge that worms were involved in the transmission pathway for LF had a mean acceptability score 1.39 points higher (p = 0.0070) than their counterparts who did not. Those who indicated that mosquitoes were involved in transmission were less accepting, scoring 2.32 (p = 0.0191) points lower, as were those who indicated other options (-3.19; p = 0.0081), or did not know (-2.30; p = 0.0277). Opinions on the existence of asymptomatic LF were associated with higher acceptability scores, whether the respondent indicated that it did (0.87; p = 0.0467) or did not (1.25; p = 0.0217) exist, compared with those who responded maybe (0.01; p = 0.9830), were unsure or didn’t know (referent). Individuals who responded with “neutral” to their opinion that MDA is important for community health had higher acceptability (3.03; p = < 0.0001) than those who did not know if MDA was important. Individuals who understood that you should take LF pills even if you are not sick had mean acceptability scores of 2.94 (p = 0.0003) higher over those who did not know. Systematic non-compliance (or never treated) was also associated with acceptability. Individuals who had taken the treatment once (2.20; p < 0.0001) or more than once (2.33; p < 0.0001) had higher acceptability scores than those who reported that they have never been treated. Education, age, sex, and primary source of income were not associated with acceptability in this study.

**Table 2:** Univariable and adjusted linear regression predicting composite acceptability score in the primary study population.

Variable		Univariable Model			Adjusted Model		
	Freq.	Coeff.	(95% CI)	pvalue	Coeff.	(95% CI)	pvalue
**Region**							
Region III	111	REF			REF		
Region IV	106	2.05	(1.10 ─ 2.99)	<0.0001	1.22	(0.39 ─ 2.04)	0.0038
Region V	116	4.65	(3.72 ─ 5.57)	<0.0001	3.95	(3.12 ─ 4.78)	<0.0001
Region X	57	2.42	(1.29 ─ 3.56)	<0.0001	1.40	(0.42 ─ 2.39)	0.0052
**Sex**							
Male	106	REF			─	─	─
Female	284	0.96	(0.08 ─ 1.84)	0.0334			
**Age**							
18–25	63	REF					
26–35	71	1.01	(-0.33 ─ 2.35)	0.1410			
36–45	84	0.33	(-0.96 ─ 1.62)	0.6175	─	─	─
46–55	84	0.66	(-0.63 ─ 1.96)	0.3150			
56 +	88	0.99	(-0.29 ─ 2.27)	0.1281			
**Education level attained**							
Primary School	99	REF					
Secondary school	244	-1.12	(-2.04 ─ -0.20)	0.0170	─	─	─
College / University	47	-0.35	(-1.72 ─ 1.02)	0.6188			
**Primary source of income**							
Daily Laborer	67	REF					
Fishing or Farming (Agriculture)	32	2.39	(0.75 ─ 4.03)	0.0043			
Small Scale Enterprise	23	0.30	(-1.78 ─ 2.39)	0.7748	─	─	─
Private Employment	87	0.41	(-1.16 ─ 1.99)	0.6070			
Government / Civil servant	51	1.73	(0.01 ─ 3.45)	0.0493			
Other	130	0.53	(-0.97 ─ 2.04)	0.4895			
**Self-rated understanding of LF**							
No knowledge	79	REF					
Some knowledge	109	1.85	(0.72 ─ 2.97)	0.0013			
Average knowledge	92	1.05	(-0.12 ─ 2.22)	0.0777	─	─	─
Good knowledge	53	2.19	(0.84 ─ 3.54)	0.0015			
Very good knowledge	57	2.52	(1.20 ─ 3.85)	0.0002			
**Mechanism of transmission** *							
Worms	38	2.45	(1.14 ─ 3.75)	0.0002	1.39	(0.38 ─ 2.39)	0.0070
Mosquitos	331	1.57	(0.48 ─ 2.66)	0.0048	-2.32	(-4.27 ─ -0.38)	0.0191
Water	26	0.49	(-1.09 ─ 2.08)	0.5398	─	─	─
Hereditary	1	3.08	(-4.71 ─ 10.88)	0.4379	─	─	─
Other	8	-4.01	(-6.76 ─ -1.25)	0.0044	-3.19	(-5.55 ─ -0.83)	0.0081
Don’t know	51	-1.78	(-2.94 ─ -0.63)	0.0025	-2.30	(-4.34 ─ -0.25)	0.0277
**Believes LF to be asymptomatic**							
Yes	208	1.70	(0.70 ─ 2.71)	0.0009	0.87	(0.01 ─ 1.72)	0.0467
Maybe	53	-0.23	(-1.58 ─ 1.11)	0.7336	0.01	(-1.07 ─ 1.09)	0.9830
No	51	2.32	(0.95 ─ 3.68)	0.0009	1.25	(0.18 ─ 2.33)	0.0217
Unsure/Don’t know	78	REF			REF		
**Perception of number of people in village with LF**							
None	119	2.47	(1.58 ─ 3.35)	<0.0001			
Few	46	1.05	(-0.19 ─ 2.28)	0.0980			
Some	20	0.44	(-1.33 ─ 2.21)	0.6263	─	─	─
Lots	12	2.01	(-0.23 ─ 4.24)	0.0786			
Many	13	1.55	(-0.60 ─ 3.70)	0.1580			
Don’t know	180	REF					
**Personal concern about LF**							
No, not at all	10	0.13	(-2.72 ─ 2.98)	0.9270			
No, not really	20	0.58	(-1.73 ─ 2.90)	0.6219			
Maybe	34	3.92	(1.86 ─ 5.98)	0.0002	─	─	─
Yes, a bit	124	3.25	(1.50 ─ 5.00)	0.0003			
Yes, definitely	181	3.98	(2.27 ─ 5.69)	<0.0001			
Don’t know	21	REF					
**Number of times taken treatment**							
Never	75	REF			REF		
Once	94	3.58	(2.50 ─ 4.66)	<0.0001	2.20	(1.23 ─ 3.17)	<0.0001
Two or more times	221	4.67	(3.74 ─ 5.60)	<0.0001	2.33	(1.46 ─ 3.20)	<0.0001
**Importance of MDA for community health**							
Not Important	15	4.33	(1.72 ─ 6.95)	0.0012	1.40	(-0.37 ─ 3.16)	0.1203
Neutral	45	4.58	(2.44 ─ 6.71)	<0.0001	3.30	(1.69 ─ 4.91)	<0.0001
Important	315	7.05	(5.16 ─ 8.94)	<0.0001	1.90	(-0.51 ─ 4.31)	0.1230
Don’t know	15	REF			REF		
**Take LF pills even if not sick**							
Yes	336	6.45	(4.97 ─ 7.93)	<0.0001	2.94	(1.35 ─ 4.53)	0.0003
No	60	3.14	(1.22 ─ 5.06)	0.0014	1.42	(-0.37 ─ 3.21)	0.1203
Don’t know	24	REF			REF		

* Mechanism of transmission was indicated through a multiple-response category. Regression estimates show indication vs no indication.

### Sensitivity analysis of the acceptability model

Due to almost 40% missing data on the acceptability score response variables in Region X (as opposed to <1% missing in all other regions) we conducted a sensitivity analysis to assess the potential for bias (S1 Table). Our sensitivity model removed the remaining responses from Region X and examined only data from the more complete region profiles. The same variables and methods were used in the construction of both models. The final sensitivity model demonstrated that our primary model was robust to the missing data. Only affirmative Knowledge of Asymptomatic LF with 0.80 (p = 0.0994) and high Personal Concern about LF 1.75 (p = 0.0343) had changes to variable significance, with only minor differences from the final model. Sex improved model fit in the sensitivity model but was not significant: 0.58 (p = 0.1069).

### Regional analysis of acceptability

High degrees of variation in participants’ self-reported understanding about LF were observed across the four regions (see Table 1). Surveyed community members in Regions III and V self-reported lower levels of knowledge, with 26.1% of participants in Region III and 25.9% of participants in Region V reporting that they had “no knowledge” of the disease. The trends observed in this self-reported measure of understanding were similarly observed in participants’ knowledge of the mechanism of LF transmission. Greater proportions of survey participants from Regions IV and X (90.6% and 93.0%, respectively) identified “mosquitoes” as the way LF transmits from person to person, compared to those from Regions III and V (79.3% and 81.0%, respectively; p = 0.0220). Participants’ understanding of the asymptomatic nature of LF also varied by region (p = 0.0002). In Region IV, 63.2% of participants understood that one could be infected with LF and not exhibit symptoms, compared to 56.1%, 51.4%, and 44.8% of participants in Regions X, III, and V, respectively.

Participants’ perceptions of the burden of LF in their communities also appeared to vary by region. A large proportion of participants in each region reported not knowing how many people in their village had LF. Only survey respondents from Region IV appeared to identify LF as a pervasive issue in their community, with 18.8% of respondents reporting there were “quite a lot” or “many” people in their village with LF. Participants’ personal concern about LF also varied by region. Community members from Region IV appeared to be the most concerned, with 59.4% of respondents indicating that they were “definitely” concerned about LF. The proportion of survey respondents that reported being “definitely” concerned in Regions III, V, and X were 51.4%, 37.1%, and 31.6%, respectively.

Due to the prominence of region as a significant predictor of treatment acceptability in the aggregate model, as well as the high degree of variation in key variables across region, we constructed additional linear regression models to investigate correlates of treatment acceptability within each region as subgroup analyses (Table 3). Acceptability was not explained uniformly across the four regions by any single variable. The most commonly impactful variable was systematic compliance (or never treated) with those having participated in the MDA at least once being significantly more accepting than those who had not in Regions III, IV, and V by 1.96 (p = 0.0219), 3.43 (p < 0.0001), and 4.09 (p = 0.0034) points respectively. Knowledge of asymptomatic LF was significantly associated with acceptability in Region III by 1.74 points (p = 0.0146) and Region IV with 2.34 points (p = 0.0019) compared to those who were unsure. This closely tracks the knowledge that participants should take LF pills even when they are not symptomatic: those who answered yes in Region III were 3.91 (p = 0.0003) points higher on average compared to those who don’t know, and Region IV participants who answered yes scored 1.83 (p = 0.0346) points higher than those who responded no. Age was significantly associated with acceptability in Regions III and X, although the age groups of concern were in disagreement. Perceived importance of MDA for community health was strongly associated with acceptability in Regions IV and V, showing 3.80 (p < 0.0001) and 2.42 (p = 0.0006) point increases, respectively. Importance of the MDA for community health was also retained in the final model in Region III for model fit. Sex was important in Region V only, with females having higher acceptability scores by 1.53 (p = 0.0452) points. Level of education, primary source of income, and perception of LF prevalence in the village were not significant in any region, adjusting for other covariates.

**Table 3:** Univariable and adjusted linear regression predicting composite acceptability score in regional subsets of the primary study population.

Variable	Region III Adjusted Model			Region IV Adjusted Model			Region V Adjusted Model			Region X Adjusted Model		
	Coeff.	(95% CI)	pvalue	Coeff.	(95% CI)	pvalue	Coeff.	(95% CI)	pvalue	Coeff.	(95% CI)	pvalue
**Sex**												
Male	─	─	─	─	─	─	REF			─	─	─
Female							1.53	(0.03 ─ 3.02)	0.0452			
**Age**												
18–25	REF									REF		
26–35	1.49	(-0.11 ─ 3.09)	0.0681							-3.12	(-5.44 ─ -0.81)	0.0082
36–45	1.45	(0.01 ─ 2.89)	0.0477	─	─	─	─	─	─	-2.57	(-4.63 ─ -0.52)	0.0142
46–55	1.36	(-0.14 ─ 2.85)	0.0747							-0.85	(-2.95 ─ 1.24)	0.4250
56 +	2.23	(0.67 ─ 3.78)	0.0050							-0.34	(-2.45 ─ 1.77)	0.7505
**Self-rated understanding of LF**												
No knowledge				REF						REF		
Some knowledge				-0.96	(-2.68 ─ 0.76)	0.2729				4.76	(0.97 ─ 8.55)	0.0138
Average knowledge	─	─	─	-2.11	(-3.67 ─ -0.54)	0.0082	─	─	─	3.38	(-0.52 ─ 7.28)	0.0895
Good knowledge				-2.26	(-4.13 ─ -0.40)	0.0174				2.82	(-1.01 ─ 6.66)	0.1493
Very good knowledge				-0.64	(-2.27 ─ 0.99)	0.4405				5.96	(1.81 ─ 10.11)	0.0049
**Mechanism of transmission** ‡												
Worms							2.50	(0.92 ─ 4.08)	0.0019			
Mosquitos	─	─	─	-1.99	(-4.08 ─ 0.10)	0.0614				─	─	─
Don’t know				-4.60	(-7.81 ─ -1.39)	0.0050						
**Believes LF to be asymptomatic**												
Yes	1.74	(0.34 ─ 3.13)	0.0146	2.34	(0.87 ─ 3.81)	0.0019						
Maybe	0.85	(-0.68 ─ 2.39)	0.2762	1.96	(-0.48 ─ 4.41)	0.1161	─	─	─	─	─	─
No	1.11	(-1.23 ─ 3.44)	0.3536	3.33	(1.56 ─ 5.09)	0.0002						
Unsure/Don’t know	REF			REF								
**Number of times taken treatment**												
Never	REF			REF			REF					
Once	1.96	(0.28 ─ 3.65)	0.0219	3.43	(2.00 ─ 4.86)	<0.0001	4.09	(1.35 ─ 6.82)	0.0034	─	─	─
Two or more times	2.16	(0.67 ─ 3.64)	0.0045	1.45	(0.24 ─ 2.67)	0.0189	4.65	(2.62 ─ 6.67)	<0.0001			
**Importance of MDA for community health**												
Not Important	*			REF			REF					
Neutral	-1.24	(-3.56 ─ 1.09)	0.2965	†			†			─	─	─
Important	0.74	(-1.63 ─ 3.11)	0.5408	3.80	(2.15 ─ 5.44)	<0.0001	2.42	(1.04 ─ 3.80)	0.0006			
Don’t know	REF			*			†					
**Take LF pills even if not sick**												
Yes	3.91	(1.77 ─ 6.05)	0.0003	1.83	(0.13 ─ 3.52)	0.0346						
No	2.79	(0.11 ─ 5.47)	0.0416	REF			─	─	─	─	─	─
Don’t know	REF			*								

* No data in this category.

† Collapsed into “Not important.”

‡ Mechanism of transmission was indicated through a multiple-response category. Regression estimates show indication vs no indication.

§ Education level attained, primary source of income, and perception of number of people in village with LF were included in the multivariable analyses but were not significant in any adjusted model.

### Analysis of key sex differences

Sex was associated with self-reported knowledge of LF (p = 0.0133). Of surveyed men, 27.4% reported having no knowledge about LF, compared to only 17.6% of females (Table 4). No significant differences were observed between males and females in understanding of how LF is transmitted or its asymptomatic nature. However, there were some variations between sex in the “don’t know” response to LF transmission and asymptomatic nature of LF with males reporting “don’t know” more frequently than females did. Perceptions of the burden of LF and levels of personal concern about the disease did not vary across sex, however males reported “don’t know” more frequently than females to a question about personal concern for LF. In a subset of participants who had participated in a previous MDA, there were no observed differences between how males or females felt about the number of pills they received (p = 0.3049), with males reporting more “don’t know” responses than women. Due to a lack of association with key variables, no model was constructed for sex.

**Table 4:** Descriptive analysis of key variables by sex in the primary study population.

Variable	Men (n = 106)		Women (n = 284)		Total (n = 390)		pvalue
	Frequency	%	Frequency	%	Frequency	%	
Self-rated understanding of LF							
No knowledge (1)	29	27.4%	50	17.6%	79	20.3%	0.0133
A little (2)	26	24.5%	83	29.2%	109	27.9%	
Average (3)	32	30.2%	60	21.1%	92	23.6%	
Good (4)	8	7.5%	45	15.8%	53	13.6%	
Very good (5)	11	10.4%	46	16.2%	57	14.6%	
Mechanism of LF transmission †							
Worms	7	6.6%	31	10.9%	38	9.7%	0.2015
Mosquitoes	84	79.2%	247	87.0%	331	84.9%	0.0582
Water	6	5.7%	20	7.0%	26	6.7%	0.6265
Hereditary	1	0.9%	0	0.0%	1	0.3%	*0.2718
Other	5	4.7%	3	1.1%	8	2.1%	*0.0371
Don’t Know	16	15.1%	35	12.3%	51	13.1%	0.4703
Believes LF to be asymptomatic							
Yes	49	46.2%	159	56.0%	208	53.3%	0.3758
Maybe	16	15.1%	37	13.0%	53	13.6%	
No	17	16.0%	34	12.0%	51	13.1%	
Don’t know	24	22.6%	54	19.0%	78	20.0%	
Perception of number of people in village with LF							
None (1)	36	34.0%	83	29.2%	119	30.5%	*0.7622
Few (2)	10	9.4%	36	12.7%	46	11.8%	
Some (3)	4	3.8%	16	5.6%	20	5.1%	
Quite a lot (4)	3	2.8%	9	3.2%	12	3.1%	
Many (5)	5	4.7%	8	2.8%	13	3.3%	
Don’t know	48	45.3%	132	46.5%	180	46.2%	
Personal concern about LF							
No, not at all (1)	4	3.8%	6	2.1%	10	2.6%	*0.2557
Not really (2)	8	7.5%	12	4.2%	20	5.1%	
Maybe (3)	10	9.4%	24	8.5%	34	8.7%	
Yes, a bit (4)	30	28.3%	94	33.1%	124	31.8%	
Yes, definitely (5)	45	42.5%	136	47.9%	181	46.4%	
Don’t Know	9	8.5%	12	4.2%	21	5.4%	
Feeling about the number of pills received ‡							
Very happy	12	16.4%	48	21.2%	60	20.1%	*0.3049
Happy	16	21.9%	70	31.0%	86	28.8%	
Neutral	33	45.2%	78	34.5%	111	37.1%	
Unhappy	5	6.8%	16	7.1%	21	7.0%	
Very unhappy	5	6.8%	12	5.3%	17	5.7%	
Don’t know	2	2.7%	2	0.9%	4	1.3%	
Number of times taken treatment							
Never	23	21.7%	52	18.3%	75	19.2%	0.4501
≥ One time	83	78.3%	232	81.7%	315	80.8%	

* Denotes Fisher’s Exact Test.

† Mechanism of transmission was indicated through a multiple-response category. Percentages may exceed 100%.

‡ Question was only asked to those who said they remember the previous mass drug administration.

### Analysis of patterns of never treated or systematic non-compliance

Systematic non-compliance with treatment is a self-reported measure whereby the participant reported that they have never taken treatment for LF. Survey participants reported high compliance with the most recent MDA and previous rounds of MDA (Table 5). Across all regions, 19.2% of participants self-identified as having never been treated during an MDA. Region IV had the greatest proportion of systematic non-compliers, with 28.3% of community members saying that they had never taken any treatment for LF during MDA. Some knowledge indicators differed between individuals who had never been treated and those who reported to have been treated before (either once or more than once). Self-rated understanding of LF was associated with ever participating in an MDA (p < 0.0001). Over 42% of those who had never participated in MDA reported having “no knowledge” of LF, compared to only 14.9% of those who had previously participated in MDA. A greater proportion of those who had previously participated in MDA also understood that the disease was spread by mosquitoes compared to those who had never participated (87.6% to 73.3%, p = 0.0019). Similarly, a greater proportion of those who had never participated in MDA reported not knowing how LF was transmitted compared to those who had previously participated (24.0% to 10.5%, p = 0.0018). Those who reported having “no knowledge” of LF did not know how LF was spread and who were not as personally concerned about the disease also tended to be never treated. When asked if the respondent was personally concerned about LF, 20% of the never treated individuals reported they were not concerned, versus only 4.8% of individuals having been treated at least once. A higher proportion of individuals who reported “don’t know” to their level of concern about LF were found to have never been treated, as opposed to those who had taken the treatment once, or more than once. Community members who had never been treated during MDA did not perceive the LF burden in their communities differently than those who had previously participated (p = 0.5726).

**Table 5:** Descriptive analysis of key demographic and knowledge variables across participants’ historical compliance with MDA in the primary study population.

Variable	Never Treated		Treated ≥ Once		Total (n = 390)		pvalue
	Frequency	%	Frequency	%	Frequency	%	
Region							
Region III	30	40.0%	81	25.7%	111	28.5%	<0.0001
Region IV	30	40.0%	76	24.1%	106	27.2%	
Region V	12	16.0%	104	33.0%	116	29.7%	
Region X	3	4.0%	54	17.1%	57	14.6%	
Sex							
Male	23	30.7%	83	26.3%	106	27.2%	0.4501
Female	52	69.3%	232	73.7%	284	72.8%	
Age							
18–25	12	16.0%	51	16.2%	63	16.2%	0.6374
26–35	18	24.0%	53	16.8%	71	18.2%	
36–45	16	21.3%	68	21.6%	84	21.5%	
46–55	13	17.3%	71	22.5%	84	21.5%	
56 +	16	21.3%	72	22.9%	88	22.6%	
Level of education							
No school at all	2	2.7%	5	1.6%	7	1.8%	*0.9123
Completed primary school	15	20.0%	77	24.4%	92	23.6%	
Completed middle school	7	9.3%	30	9.5%	37	9.5%	
Completed secondary school	42	56.0%	165	52.4%	207	53.1%	
Completed college/university	9	12.0%	36	11.4%	45	11.5%	
Other	0	0.0%	2	0.6%	2	0.5%	
Self-rated understanding of LF							
No knowledge	32	42.7%	47	14.9%	79	20.3%	<0.0001
Some knowledge	16	21.3%	93	29.5%	109	27.9%	
Average knowledge	13	17.3%	79	25.1%	92	23.6%	
Good knowledge	2	2.7%	51	16.2%	53	13.6%	
Very good knowledge	12	16.0%	45	14.3%	57	14.6%	
Mechanism of LF transmission †							
Worms	7	9.3%	31	9.8%	38	9.7%	0.8939
Mosquitoes	55	73.3%	276	87.6%	331	84.9%	0.0019
Water	3	4.0%	23	7.3%	26	6.7%	0.3029
Hereditary	0	0.0%	1	0.3%	1	0.3%	*0.8077
Other	3	4.0%	5	1.6%	8	2.1%	*0.1846
Don’t Know	18	24.0%	33	10.5%	51	13.1%	0.0018
Believes LF to be asymptomatic							
Yes	33	44.0%	175	55.6%	208	53.3%	0.3178
Maybe	11	14.7%	42	13.3%	53	13.6%	
No	12	16.0%	39	12.4%	51	13.1%	
Don’t know	19	25.3%	59	18.7%	78	20.0%	
Perception of number of people in village with LF							
None	18	24.0%	101	32.1%	119	30.5%	*0.5726
Few	8	10.7%	38	12.1%	46	11.8%	
Some	4	5.3%	16	5.1%	20	5.1%	
Lots	4	5.3%	8	2.5%	12	3.1%	
Many	2	2.7%	11	3.5%	13	3.3%	
Don’t know	39	52.0%	141	44.8%	180	46.2%	
Personal concern about LF							
No, not at all	6	8.0%	4	1.3%	10	2.6	*0.0004
No, not really	9	12.0%	11	3.5%	20	5.1%	
Maybe	4	5.3%	30	9.5%	34	8.7%	
Yes, a bit	21	28.0%	103	32.7%	124	31.8%	
Yes, definitely	28	37.3%	153	48.6%	181	46.4%	
Don’t know	7	9.3%	14	4.4%	21	5.4%	
Importance of MDA for community health							
Not Important	7	9.3%	8	2.5%	15	3.8%	*<0.0001
Neutral	12	16.0%	33	10.5%	45	11.5%	
Important	44	58.7%	271	86.0%	315	80.8%	
Don’t know	12	16.0%	3	1.0%	15	3.8%	
Take LF pills even if not sick							
Yes	46	61.3%	290	92.1%	336	86.2%	<0.0001
No	13	17.3%	17	5.4%	30	7.7%	
Don’t know	16	21.3%	8	2.5%	24	6.2%	

* Denotes Fisher’s Exact Test.

† Mechanism of transmission was indicated through a multiple-response category. Percentages may exceed 100%.

Multivariable analysis (Table 6) was conducted to understand the relationship between the never treated during MDA compared to those who had been treated at least once (n = 390). The odds of never treatment during MDA for LF were higher in Regions III (Adjusted Odds Ratio 6.97; p = 0.0076) and IV (AOR 13.51; p = 0.0002) compared to Region X. Those who did not know the mode of transmission for LF were nearly three times as likely to have never been treated during MDA (AOR 2.76; p = 0.0404) compared to those who indicated some theory of transmission. Having no knowledge of asymptomatic LF was associated with lower likelihood of treatment (AOR 3.34; p = 0.0417) compared to those who were unsure. Similarly, those who expressed no personal concern about LF were more likely to have never been treated during MDA than those who “don’t know” if they were concerned (AOR 7.15; p = 0.0401). Individuals who reported that MDA was important for community health (AOR 0.10; p = 0.0140) or who were neutral (AOR 0.16; p = 0.0711) in their response also were less likely to have never been treated than those who reported “don’t know.” Finally, individuals who believed that you should take the LF pills even if you are not sick were less likely to have never been treated (AOR 0.14; p = 0.0094) than individuals who responded, “don’t know” to this statement. Age, sex, level of education, primary source of income, perceived understanding of lymphatic filariasis, and perception of LF prevalence in their village were not related to self-reported history of treatment.

**Table 6:** Univariable and adjusted logistic regression predicting odds of systematic non-compliance (never treated) with MDA in the primary study population.

Variable		Univariable Model			Adjusted Model		
	Freq.	OR	(95% CI)	pvalue	AOR	(95% CI)	pvalue
**Region**							
Region III	111	6.67	(1.94 ─ 22.94)	0.0026	6.97	(1.68 ─ 29.00)	0.0076
Region IV	106	7.11	(2.06 ─ 24.48)	0.0019	13.51	(3.40 ─ 53.67)	0.0002
Region V	116	2.08	(0.56 ─ 7.68)	0.2731	1.95	(0.42 ─ 8.97)	0.3926
Region X	57	REF					
**Sex**							
Male	106	1.24	(0.71 ─ 2.15)	0.4506	─	─	─
Female	284	REF					
**Age**							
18–25	63	1.06	(0.46 ─ 2.43)	0.8926			
26–35	71	1.53	(0.71 ─ 3.27)	0.2747			
36–45	84	1.06	(0.49 ─ 2.28)	0.8840	─	─	─
46–55	84	0.82	(0.37 ─ 1.84)	0.6360			
56 +	88	REF					
**Education level attained**							
Primary School	99	REF					
Secondary school	244	1.21	(0.66 ─ 2.23)	0.5360	─	─	─
College / University	47	1.14	(0.47 ─ 2.80)	0.7706			
**Primary source of income**							
Daily Laborer	67	REF					
Fishing or Farming (Agriculture)	32	2.52	(0.89 ─ 7.15)	0.0821			
Small Scale Enterprise	23	1.36	(0.37 ─ 4.91)	0.6421			
Private Employment	87	1.80	(0.76 ─ 4.29)	0.1837	─	─	─
Government / Civil servant	51	1.20	(0.43 ─ 3.36)	0.7301			
Other	130	1.61	(0.71 ─ 3.67)	0.2562			
**Self-rated understanding of LF**							
No/Some knowledge	188	2.35	(1.23 ─ 4.50)	0.0099			
Average knowledge	92	1.13	(0.50 ─ 2.54)	0.7705	─	─	─
Good/Very good knowledge	110	REF					
**Mechanism of transmission** *							
Worms	38	0.94	(0.40 ─ 2.23)	0.8940	─	─	─
Mosquitos	276	0.39	(0.21 ─ 0.72)	0.0025	─	─	─
Don’t know	51	2.70	(1.42 ─ 5.12)	0.0024	2.76	(1.05 ─ 7.26)	0.0404
**Believes LF to be asymptomatic**							
Yes	208	0.59	(0.31 ─ 1.11)	0.0996	1.23	(0.46 ─ 3.25)	0.6795
Maybe	53	0.81	(0.35 ─ 1.89)	0.6302	1.06	(0.32 ─ 3.50)	0.9217
No	51	0.96	(0.42 ─ 2.19)	0.9141	3.34	(1.05 ─ 10.66)	0.0417
Unsure/Don’t know	78	REF					
**Perception of number of people in village with LF**							
None/Few	165	0.68	(0.39 ─ 1.17)	0.1624			
Some	20	0.90	(0.29 ─ 2.86)	0.8634	─	─	─
Lots/Many	25	1.14	(0.43 ─ 3.05)	0.7918			
Don’t know	180	REF					
**Personal concern about LF**							
No, not at all/No, not really	30	2.00	(0.63 ─ 6.35)	0.2397	7.15	(1.09 ─ 46.77)	0.0401
Maybe	34	0.27	(0.07 ─ 1.06)	0.0610	0.83	(0.11 ─ 6.03)	0.8519
Yes, a bit/Yes, definitely	305	0.38	(0.15 ─ 1.00)	0.0493	2.29	(0.41 ─ 12.88)	0.3458
Don’t know	21	REF					
**Importance of MDA for community health**							
Not Important	15	0.22	(0.04 ─ 1.11)	0.0662	1.23	(0.14 ─ 11.08)	0.8565
Neutral	45	0.09	(0.02 ─ 0.38)	0.0010	0.16	(0.02 ─ 1.17)	0.0711
Important	315	0.04	(0.01 ─ 0.15)	<0.0001	0.10	(0.02 ─ 0.63)	0.0140
Don’t know	15	REF					
**Take LF pills even if not sick**							
Yes	336	0.08	(0.03 ─ 0.20)	<0.0001	0.14	(0.03 ─ 0.62)	0.0094
No	30	0.38	(0.13 ─ 1.17)	0.0908	0.54	(0.11 ─ 2.73)	0.4597
Don’t know	24	REF					

* Mechanism of transmission was indicated through a multiple-response category. Regression estimates show indication vs no indication.

### MDA participant subset analysis

Community members who remembered the most recent distribution (n = 289) were asked a series of questions relating to their experience with the MDA (Table 7). Most of these respondents (87.2%) stated that other members of their household also participated in the MDA. Overall, 49.0% reported feeling “happy” or “very happy” about the number of pills they received, while 36.9% reported feeling “neutral.” Some regional variation was observed in this variable. Regions III and V had the largest proportions of community members reporting that they were “happy” or “very happy” with the number of pills they received (55.1% and 58.2%), while Region X had the smallest (28.9%). Region X also had the greatest proportion of survey respondents indicating they were “unhappy” or “very unhappy” with the number of pills they received (23.1%) compared to the other regions (5.0%, 14.5%, and 12.3% in Regions III, VI, and V, respectively). Some regional variation was observed.

**Table 7:** Descriptive analysis of key variables in a subset of the primary study population who have previously participated in an MDA.

Variable	Region III (n = 80)		Region IV (n = 68)		Region V (n = 98)		Region X (n = 52)		Total (n = 298)	
	Frequency	%	Frequency	%	Frequency	%	Frequency	%	Frequency	%
Other members of the household took pills at the same time										
Yes	68	85.0%	61	89.7%	95	96.9%	36	69.2%	260	87.2%
No	3	3.8%	4	5.9%	2	2.0%	8	15.4%	17	5.7%
Don’t know	9	11.3%	3	4.4%	1	1.0%	8	15.4%	21	7.0%
Feeling about the number of pills received										
Very happy	25	31.3%	17	25.0%	14	14.3%	4	7.7%	60	20.1%
Happy	19	23.8%	13	19.1%	43	43.9%	11	21.2%	86	28.9%
Neutral	31	38.8%	28	41.2%	29	29.6%	22	42.3%	110	36.9%
Unhappy	2	2.5%	6	8.8%	8	8.2%	5	9.6%	21	7.0%
Very unhappy	2	2.5%	4	5.9%	4	4.1%	7	13.5%	17	5.7%
Don’t know/Unsure	1	1.3%	0	0.0%	0	0.0%	3	5.8%	4	1.3%
Description of Information regarding the LF treatment										
Not useful	5	6.3%	1	1.5%	0	0.0%	2	3.8%	8	2.7%
A little useful	10	12.5%	0	0.0%	2	2.0%	2	3.8%	14	4.7%
Neutral	26	32.5%	4	5.9%	4	4.1%	6	11.5%	40	13.4%
Useful	11	13.8%	33	48.5%	34	34.7%	18	34.6%	96	32.2%
Very useful	24	30.0%	28	41.2%	58	59.2%	18	34.6%	128	43.0%
Don’t know/Didn’t receive info	4	5.0%	2	2.9%	0	0.0%	6	11.5%	12	4.0%
Opinion on LF drug safety										
Very dangerous	0	0.0%	0	0.0%	0	0.0%	0	0.0%	0	0.0%
Dangerous	1	1.3%	2	2.9%	3	3.1%	1	1.9%	7	2.3%
Neutral	17	21.3%	6	8.8%	8	8.2%	7	13.5%	38	12.8%
Safe	13	16.3%	13	19.1%	13	13.3%	26	50.0%	65	21.8%
Very safe	47	58.8%	44	64.7%	73	74.5%	13	25.0%	177	59.4%
Don’t know	2	2.5%	3	4.4%	1	1.0%	5	9.6%	11	3.7%

We used this subset of participants who had taken the LF treatment previously to explore the attitudes related to compliance with the 2018 DEC/Albendazole MDA. As with the acceptability model, region was an important factor in compliance with treatment (Table 8). Those in Regions IV, V, and X had higher acceptability scores than individuals in Region III (1.16 (p = 0.0204), 3.27 (p < 0.0001), and 1.47 (p = 0.0045) points higher, respectively). Individuals who understood worms were implicated in LF transmission also scored 1.14 (p = 0.0342) points higher. Those who perceived that there was no LF in their village had higher scores (1.03; p = 0.0090) compared to those who did not know about LF prevalence in the community. Finally, results indicated that those who believed that the treatment was very safe were more accepting (1.59; p = 0.0695) than those who did not know if the LF drugs were safe or not.

**Table 8:** Univariable and adjusted linear regression predicting composite acceptability score in a subset of the primary study population who have previously participated in an MDA.

Variable		Univariable Model			Adjusted Model		
	Freq.	Coeff.	(95% CI)	pvalue	Coeff.	(95% CI)	pvalue
**Region**							
Region III	80	REF			REF		
Region IV	68	1.24	(0.29 ─ 2.19)	0.0105	1.16	(0.18 ─ 2.14)	0.0204
Region V	98	4.04	(3.17 ─ 4.90)	<0.0001	3.27	(2.41 ─ 4.14)	<0.0001
Region X	52	1.13	(0.10 ─ 2.15)	0.0309	1.47	(0.45 ─ 2.48)	0.0045
**Sex**							
Male	73	REF					
Female	225	0.96	(0.08 ─ 1.84)	0.0330	─	─	─
**Age**							
18–25	48	REF					
26–35	50	0.86	(-0.47 ─ 2.18)	0.2056			
36–45	66	0.66	(-0.58 ─ 1.91)	0.2958	─	─	─
46–55	66	0.98	(-0.26 ─ 2.22)	0.1218			
56 +	68	0.88	(-0.36 ─ 2.11)	0.1650			
**Education level attained**							
Primary School	78	REF					
Secondary school	184	-1.19	(-2.06 ─ -0.31)	0.0082	─	─	─
College / University	36	-1.27	(-2.58 ─ 0.04)	0.0567			
**Primary source of income**							
Daily Laborer	55	REF					
Fishing or Farming (Agriculture)	22	2.86	(1.27 ─ 4.46)	0.0004			
Small Scale Enterprise	18	0.78	(-1.23 ─ 2.79)	0.4448	─	─	─
Private Employment	63	0.12	(-1.45 ─ 1.68)	0.8843			
Government / Civil servant	41	1.20	(-0.47 ─ 2.87)	0.1580			
Other	99	0.88	(-0.61 ─ 2.37)	0.2447			
**Self-rated understanding of LF**							
No knowledge	41	REF					
Some knowledge	91	-0.16	(-1.37 ─ 1.06)	0.8015			
Average knowledge	77	-1.40	(-2.65 ─ -0.15)	0.0282	─	─	─
Good knowledge	45	-0.42	(-1.82 ─ 0.97)	0.5538			
Very good knowledge	44	0.42	(-0.99 ─ 1.82)	0.5621			
**Mechanism of transmission** †							
Worms	30	2.19	(0.95 ─ 3.43)	0.0006	1.14	(0.08 ─ 2.19)	0.0342
Mosquitos	262	-0.06	(-1.23 ─ 1.11)	0.9140	─	─	─
Water	22	-0.46	(-1.91 ─ 1.00)	0.5390	─	─	─
Hereditary	1	*					
Other	5	-4.10	(-7.03 ─ -1.17)	0.0061	-2.53	(-5.15 ─ 0.09)	0.0584
Don’t know	30	0.11	(-1.15 ─ 1.38)	0.8619			
**Believes LF to be asymptomatic**							
Yes	168	0.32	(-0.69 ─ 1.34)	0.5309			
Maybe	42	-1.17	(-2.50 ─ 0.16)	0.0842	─	─	─
No	35	1.63	(0.23 ─ 3.03)	0.0221			
Unsure/Don’t know	53	REF					
**Perception of number of people in village with LF**							
None	98	2.22	(1.39 ─ 3.06)	<0.0001	1.03	(0.26 ─ 1.80)	0.0090
Few	37	0.27	(-0.90 ─ 1.44)	0.6507	0.25	(-0.81 ─ 1.31)	0.6463
Some	16	0.31	(-1.35 ─ 1.98)	0.7128	-0.25	(-1.70 ─ 1.19)	0.7299
Lots	8	1.25	(-1.04 ─ 3.54)	0.2844	0.37	(-1.62 ─ 2.37)	0.7148
Many	8	0.63	(-1.66 ─ 2.91)	0.5925	0.38	(-1.72 ─ 2.48)	0.7245
Don’t know	131	REF			REF		
**Personal concern about LF**							
No, not at all/No, not really	14	3.11	(0.56 ─ 5.65)	0.0166	3.11	(0.56 ─ 5.65)	0.0166
Maybe	27	3.53	(1.28 ─ 5.77)	0.0021	3.53	(1.28 ─ 5.77)	0.0021
Yes, a bit	99	2.32	(0.34 ─ 4.29)	0.0216	2.32	(0.34 ─ 4.29)	0.0216
Yes, definitely	146	2.75	(0.81 ─ 4.69)	0.0055	2.75	(0.81 ─ 4.69)	0.0055
Don’t know	12	REF			REF		
**Other members of the household took pills at the same time**							
Yes	250	-1.15	(-2.79 ─ 0.49)	0.1690			
No	17	-1.07	(-2.55 ─ 0.42)	0.1584	─	─	─
Don’t know	21	REF					
**Feeling about the number of pills received**							
Very happy	60	2.37	(-0.95 ─ 5.68)	0.1621			
Happy	86	2.25	(-1.04 ─ 5.54)	0.1796			
Neutral	110	0.90	(-2.38 ─ 4.17)	0.5915	─	─	─
Unhappy	21	0.56	(-2.95 ─ 4.06)	0.7544			
Very unhappy	17	1.51	(-2.06 ─ 5.09)	0.4057			
Don’t know/Unsure	4	REF					
**Description of Information regarding the LF treatment**							
Not useful	8	-0.25	(-3.06 ─ 2.56)	0.8615			
A little useful	14	0.54	(-1.88 ─ 2.96)	0.6644			
Neutral	40	0.28	(-1.75 ─ 2.30)	0.7901	─	─	─
Useful	96	2.00	(0.12 ─ 3.88)	0.0374			
Very useful	128	3.22	(1.36 ─ 5.08)	0.0007			
Don’t know/Didn’t receive info	12	REF					
**Opinion on LF drug safety**							
Very dangerous/Dangerous	7	0.30	(-2.70 ─ 3.30)	0.8453	0.00	(-2.75 ─ 2.74)	0.9982
Neutral	38	-0.61	(-2.74 ─ 1.51)	0.5707	-0.65	(-2.51 ─ 1.21)	0.4931
Somewhat safe	65	0.91	(-1.11 ─ 2.94)	0.3771	0.56	(-1.19 ─ 2.31)	0.5325
Very safe	177	2.41	(0.48 ─ 4.34)	0.0143	1.59	(-0.13 ─ 3.30)	0.0695
Don’t know	11	REF			REF		
**Importance of MDA for community health**							
Not Important	5	-2.80	(-5.94 ─ 0.34)	0.0808			
Neutral	30	-1.22	(-4.16 ─ 1.71)	0.4145	─	─	─
Important	261	-2.20	(-7.64 ─ 3.24)	0.4283			
Don’t know	2	REF					
**Take LF pills even if not sick**							
Yes	277	2.70	(0.36 ─ 5.03)	0.0238			
No	13	1.75	(-1.18 ─ 4.68)	0.2416	─	─	─
Don’t know	8	REF					

* No data in this category.

† Mechanism of transmission was indicated through a multiple-response category. Regression estimates show indication vs no indication.

## Discussion

In preparation for the 2019 MDA with IDA regimen, the research team undertook a nested acceptability study within a larger sentinel mapping study to assess the coverage, compliance, and acceptability of the 2018 MDA with DEC/Albendazole in Guyana. The study findings were then used to inform the rollout of the MDA with IDA in 2019 and to provide a baseline for further data collection to assess changes in coverage, compliance, and acceptability. The survey used a mean acceptability score as the primary outcome of interest in addition to known knowledge and perception indicators. In this study, across all four regions, acceptability of the 2018 MDA was above the threshold.

Region was an important determinant for both compliance with treatment and acceptability. Variation was reported across the four regions for many key indicators. Regions III and V reported lower levels of knowledge and higher proportions of “don’t knows” for key variables. In contrast, Region IV, where the capital Georgetown is located, reported some of the highest levels of understanding about LF and its transmission despite having the highest number of noncompliers in the last MDA (2018) and highest numbers of never treated individuals (or systematic noncompliers) along with Region III. This implies that knowledge alone is not sufficient to drive high treatment coverage. These regional variations reveal the need for more tailored approaches to the planning and social mobilization activities carried out prior to MDA. While the national program can provide support and technical guidance, regional approaches can consider past population behavior *vis à vis* MDA as well as contextual specifications. Studies reporting challenges with MDA in urban areas have suggested that specific strategies are needed in these contexts, given the complex populations, increased mobility, migration and fractured social structures found in more dense populations [38–41]. With the introduction of IDA in Guyana, specific attention can be made to the lower levels of knowledge in Regions III and V, and additional and novel distribution practices can be considered in Georgetown (Region IV) that build on past experiences with MDA to improve coverage. A specific approach to identify never treated individuals during MDA by drug distribution teams should be encouraged to ensure these individuals have their questions answered and are encouraged to participate in MDA.

One of the considerations for the introduction of IDA is the increased number of pills that community members will receive with the addition of ivermectin tablets to DEC and Albendazole. While the global acceptability study did not find any association of number of pills with acceptability [23] it remains a factor that many programs would like to investigate prior to the introduction of IDA. In this Guyanese study, the number of pills in DA MDA was not an issue associated with acceptability. This finding should reassure that additional tablets will not likely be faced with resistance in the community; however, awareness and education about the rationale for the inclusion of ivermectin is recommended to encourage uptake in the community and to signal the added value of ivermectin to the treatment protocols.

Identifying the proportion of people who have never been treated during MDA is needed so that programs understand who has missed treatment over the course of MDA [42]. This information can then be used to reorient subsequent rounds, as needed, to ensure these individuals are reached with MDA. This dataset provides some insight into barriers to individual participation which could be applied operationally in Guyana. Region remains an important indicator associated with never treatment or systematic non-compliance. The relationship between those who have never been treated during MDA and acceptability is expected; e.g. those who never take LF treatment also have lower levels of acceptability. This relationship serves to validate the use of both variables as indicators of importance to understand community response to MDA. For those who have never been treated in this dataset, it is noticeable that levels of key knowledge indicators and general perception of risk for LF are lower than for those who have taken treatment in the past. The relationship is striking between those who report that they “don’t know” as opposed to those who made a choice on a question (correct or incorrect / yes or no). There were higher proportions of systematic noncompliers who reported that they “did not know” if they were concerned about LF or how the disease was spread. This may indicate indifference, lack of knowledge, or a lack of access to information.

This phenomenon carried into other analyses whereby the “don’t know” category acted differently than those who had taken a position, even a neutral position. Enumerators in this study were trained not to offer the option of “don’t know” while administering the questionnaire, to minimize the use of the “don’t know” response in the dataset. This was done so that a “don’t know” response was not given by respondents out of laziness or to please interviewers but was used to elucidate when respondents did not know the response. “Don’t know” categories in health behavior research can be problematic in analysis and can reduce validity of the findings [43]. Regardless, evidence shows that there is more nuance needed in our understanding of this response [43]. In this dataset, the interpretation of the “don’t know” category is consistent and seems to suggest that it is part of a linear progression, whereby “don’t know” represents a lower understanding or awareness compared to individuals who have formed an opinion (either affirmative or negative). For the purposes of the Guyanese program, further analysis of the “don’t know” responses may provide some direction to tailor future social mobilization activities.

A few key messages emerged that were related to acceptability, compliance, and history of compliance. They included: the need to take the treatment if not sick; the importance of MDA for community health; LF drugs are safe and; knowing about LF transmission. Some of these messages have been shown to be important in other global studies and confirm that scientific or medical knowledge alone may not be the most important in motivating behavior to comply with treatment. In Indonesia, for example, drug safety and understanding the importance of MDA for the community were shown to be associated with compliance in two districts [44]. Other studies have shown that some understanding about LF transmission is important for compliance [45–48]. Further understanding about how these messages resonate across sex, age groups and regions would help to refine upcoming health information campaigns prior to MDA delivery.

Based on these data, recommendations for the Guyana LF program suggest that a regional perspective is needed to promote acceptability, coverage, and compliance with LF treatment. The results from this study can be used to develop a more tailored approach to community mobilization, assessing gaps and building on strengths of the current activities to provide a solid awareness campaign prior to IDA rollout. The number of pills will not likely be an issue based on these data, however messaging about the addition of ivermectin will be important based on experiences from other IDA rollout countries (*personal communication A Krentel)*. The detailed analysis of those who have never been treated provides an insight into how specific messages and distribution approaches may be oriented to ensure that these individuals will be reached with the 2019 MDA with IDA. The measurements of acceptability and systematic non-compliance can also serve as a baseline from which to assess the performance of subsequent MDA rounds. With the enhanced approach to MDA and additional measures applied with the use of IDA, we should expect to see some of these never treated individuals reached for the first time in 2019. In addition, follow-up studies can assess if there is improved acceptability (or not) to the use of IDA in these regions, following the 2019 MDA.

Finally, this paper shows the value of performing applied research to assess social behavior and factors related to coverage to inform subsequent MDA rounds, particularly before the national introduction of IDA. This study was incorporated within a larger sentinel site study and so provided an opportunity to include these questions with minimal additional budget. Similar opportunities should be explored whereby a short assessment of history of treatment, acceptability and perception across key messages can be added to parasitological mapping and assessments as well as to coverage surveys. This will not only be more cost-efficient but will allow a more detailed understanding of community response to MDA so that future rounds can be reoriented, as needed. The Guyanese program applied the recommendations from this study in both rounds of IDA in 2019 and 2021. Because the data were collected only a few months prior to the 2019 MDA, there was limited time to apply all recommendations for that round. In 2021, there was more time to plan and a more regional approach to social mobilization was promoted.

### Limitations of study

Perhaps the most important limitation of the study was the missing data for Region X for the key outcome of interest, acceptability. After repeated attempts to understand why this occurred, the research team decided to move forward with the analysis and assume that there was no chance of data recovery. To account for these missing data, the team undertook a sensitivity analysis to ascertain the impact on the final acceptability model. In that model, the team was assured of the robustness of the final model which included a smaller sample from Region X, as compared to the other three regions.

Studies of this nature benefit from the added qualitative component to validate the results that arise from the survey results. In this case, timing and budgetary constraints prohibited the research team from carrying out concurrent qualitative research to understand those individual factors underpinning MDA coverage. The study team would recommend additional research to build on the findings of this study to further probe into the reasons why people refuse to take treatment over multiple rounds and into the rationale behind the “don’t know” responses, particularly to understand why there are differences across sex.

Other important limitations to the study include the over-representation of women in the study sample and the targeted nature of the Filarial Re-Mapping Survey. Given the time of the day when the data were collected, men are often outside of the home. This phenomenon has been seen in similar studies [44]. Compliance and acceptability have been shown to be highly contextual, dependent on multiple personal, social, community and health system factors. As such, we are unable to comment on whether this over-representation of women skews the study more positively or negatively. Additionally, the acceptability survey was based on a subsample of a large-scale effort to re-map and define implementation units where MDA rollout may pose particular challenges. These site characteristics (high transmission settings, cultural, geographic, or economic barriers to MDA access, etc.) contribute to concerns about the generalizability of the sample, though the aforementioned lack of a qualitative component make it difficult to assess how this may bias the results.

In a similar study we did not see an association between acceptability and infection status [23]. Regardless, inclusion of this data and that of related protective behaviors such as bednet use or window screens would have been useful to include in the analysis if the data had been available.

### Conclusion

The study showed sufficient levels of acceptability of previous MDA rounds in four regions in Guyana. Region was the most important indicator for compliance and acceptability suggesting that tailored regionally specific responses may be necessary to ensure effective coverage with MDA. The results from the study were used to inform the next two rounds of MDA (2019 and 2021) and provided a baseline level of acceptability for MDA with DA against which IDA can be measured.

## Supporting information

S1 Table Sensitivity analysis comparing the full dataset acceptability model (see Table 2) with a subset that removes remaining data from Region X. (DOCX)
