# Supplementary material for: Assessing factors influencing communities’ acceptability of mass drug administration for the elimination of lymphatic filariasis in Guyana
Source: PLoS Negl Trop Dis. 2021 Sep 20;15(9):e0009596. doi: 10.1371/journal.pntd.0009596 (PMC8452018; doi:10.1371/journal.pntd.0009596)
Supplement: S1 Table — (DOCX) [file pntd.0009596.s001.docx]

**S1 Table. Sensitivity analysis comparing the full dataset acceptability model (see Table 2) with a subset that removes remaining data from Region X.**

| **Variable** |  | **Adjusted Model** | | |  | **Adjusted Sensitivity Model** | | |
| --- | --- | --- | --- | --- | --- | --- | --- | --- |
|  | **Freq.** | **Coeff.** | **(95% CI)** | **pvalue** | **Freq.** | **Coeff.** | **(95% CI)** | **pvalue** |
| **Region** |  |  |  |  |  |  |  |  |
| Region III | 111 | REF |  |  | 111 | REF |  |  |
| Region IV | 106 | 1.22 | (0.39 ─ 2.04) | 0.0038 | 106 | 1.04 | (0.21 ─ 1.87) | 0.0146 |
| Region V | 116 | 3.95 | (3.12 ─ 4.78) | <0.0001 | 116 | 4.03 | (3.17 ─ 4.88) | <0.0001 |
| Region X | 57 | 1.40 | (0.42 ─ 2.39) | 0.0052 | 0 | * |  |  |
| **Sex** |  |  |  |  |  |  |  |  |
| Male | 106 | ─ | ─ | ─ | 90 | REF |  |  |
| Female | 284 |  |  |  | 243 | 0.58 | (-0.13 ─ 1.29) | 0.1069 |
| **Mechanism of transmission** |  |  |  |  |  |  |  |  |
| Worms | 38 | 1.39 | (0.38 ─ 2.39) | 0.0070 | 34 | 1.36 | (0.30 ─ 2.42) | 0.0120 |
| Mosquitos | 331 | -2.32 | (-4.27 ─ -0.38) | 0.0191 | 278 | -2.79 | (-4.80 ─ -0.77) | 0.0067 |
| Water | 26 | ─ | ─ | ─ | 21 | ─ | ─ | ─ |
| Hereditary | 1 | ─ | ─ | ─ | 1 | ─ | ─ | ─ |
| Other | 8 | -3.19 | (-5.55 ─ -0.83) | 0.0081 | 6 | -3.22 | (-5.92 ─ -0.51) | 0.0198 |
| Don’t know | 51 | -2.3 | (-4.34 ─ -0.25) | 0.0277 | 48 | -2.32 | (-4.43 ─ -0.22) | 0.0304 |
| **Believes LF to be asymptomatic** |  |  |  |  |  |  |  |  |
| Yes | 208 | 0.87 | (0.01 ─ 1.72) | 0.0467 | 176 | 0.80 | (-0.15 ─ 1.76) | 0.0994 |
| Maybe | 53 | 0.01 | (-1.07 ─ 1.09) | 0.9830 | 51 | -0.04 | (-1.19 ─ 1.11) | 0.9456 |
| No | 51 | 1.25 | (0.18 ─ 2.33) | 0.0217 | 39 | 1.38 | (0.18 ─ 2.58) | 0.0242 |
| Unsure/Don’t know | 78 | REF |  |  | 67 | REF |  |  |
| **Personal concern about LF** |  |  |  |  |  |  |  |  |
| No, not at all | 10 |  |  |  | 9 | -0.09 | (-2.45 ─ 2.26) | 0.9377 |
| No, not really | 20 |  |  |  | 19 | 0.98 | (-1.04 ─ 2.99) | 0.3423 |
| Maybe | 34 | ─ | ─ | ─ | 32 | 0.97 | (-0.83 ─ 2.76) | 0.2908 |
| Yes, a bit | 124 |  |  |  | 92 | 0.85 | (-0.78 ─ 2.49) | 0.3050 |
| Yes, definitely | 181 |  |  |  | 163 | 1.75 | (0.13 ─ 3.36) | 0.0343 |
| Don’t know | 21 |  |  |  | 18 | REF |  |  |
| **Number of times taken treatment** |  |  |  |  |  |  |  |  |
| Never | 75 | REF |  |  | 72 |  |  |  |
| Once | 94 | 2.20 | (1.23 ─ 3.17) | <0.0001 | 65 | 2.30 | (1.23 ─ 3.36) | <0.0001 |
| Two or more times | 221 | 2.33 | (1.46 ─ 3.20) | <0.0001 | 196 | 2.04 | (1.12 ─ 2.96) | <0.0001 |
| **Importance of MDA for community health** |  |  |  |  |  |  |  |  |
| Not Important | 15 | 1.40 | (-0.37 ─ 3.16) | 0.1203 | 15 | 0.95 | (-0.88 ─ 2.78) | 0.3080 |
| Neutral | 45 | 3.30 | (1.69 ─ 4.91) | <0.0001 | 40 | 3.03 | (1.38 ─ 4.68) | 0.0003 |
| Important | 315 | 1.90 | (-0.51 ─ 4.31) | 0.1230 | 267 | 1.31 | (-1.30 ─ 3.93) | 0.3253 |
| Don’t know | 15 | REF |  |  | 13 | REF |  |  |
| **Take LF pills even if not sick** |  |  |  |  |  |  |  |  |
| Yes | 336 | 2.94 | (1.35 ─ 4.53) | 0.0003 | 290 | 2.56 | (0.77 ─ 4.36) | 0.0052 |
| No | 60 | 1.42 | (-0.37 ─ 3.21) | 0.1203 | 22 | 1.04 | (-1.00 ─ 3.08) | 0.3163 |
| Don’t know | 24 | REF |  |  | 21 | REF |  |  |

* Category represents data omitted for the sensitivity analysis

† Age, education level attained, primary source of income, self-rated understanding of LF, and perception of the number of

people in the village with LF were included in the multivariable analyses but were not significant in either adjusted model.
